# Patterns of Feeding by Householders Affect Activity of Hedgehogs (*Erinaceus europaeus*) during the Hibernation Period

**DOI:** 10.3390/ani10081344

**Published:** 2020-08-04

**Authors:** Abigail Gazzard, Philip J. Baker

**Affiliations:** School of Biological Sciences, University of Reading, Whiteknights, Reading, Berkshire RG6 6AS, UK; p.j.baker@reading.ac.uk

**Keywords:** conservation, urban ecology, hedgehogs, *Erinaceus europaeus*, citizen science, gardens, occupancy

## Abstract

**Simple Summary:**

Urban areas are thought to represent a stronghold habitat for the West European hedgehog population in the UK. However, little is known about hibernation patterns in residential areas and if overwinter activity is influenced by any ”urban-associated” factors. We monitored hedgehog activity in gardens during the winter hibernation period of 2017–2018 using weekly presence/absence surveys. Hedgehogs were more likely to be present in gardens where householders had provided food in previous seasons or where food was supplied more regularly in a given season. Such relationships could have positive or negative effects on the survival or condition of hedgehogs across the hibernation period. Consequently, further research is needed to identify the effects of supplementary feeding on hibernation biology to help inform conservation guidelines for householders.

**Abstract:**

West European hedgehogs (*Erinaceus europaeus*) are likely to encounter unusual ecological features in urban habitats, such as anthropogenic food sources and artificial refugia. Quantifying how these affect hedgehog behaviour is vital for informing conservation guidelines for householders. We monitored hedgehog presence/absence in gardens in the town of Reading, UK, over the winter of 2017–2018 using a volunteer-based footprint tunnel survey, and collected data on garden characteristics, supplementary feeding (SF) habits, and local environmental conditions. Over a 20-week survey period, hedgehog presence was lowest between January and March. Occupancy analysis indicated that SF significantly affected hedgehog presence/absence before, during, and after hibernation. The number of nesting opportunities available in gardens, average temperatures, and daylength were also supported as important factors at different stages. In particular, our results suggest that SF could act to increase levels of activity during the winter when hedgehogs should be hibernating. Stimulating increased activity at this sensitive time could push hedgehogs into a net energy deficit or, conversely, help some individuals survive which might not otherwise do so. Therefore, further research is necessary to determine whether patterns of feeding by householders have a positive or negative effect on hedgehog populations during the hibernation period.

## 1. Introduction

Hibernation is critical for the overwinter survival of a range of vertebrate and invertebrate species [1,2,3,4]. A reduced core body temperature and lowered metabolic rate allows individuals to conserve energy during periods of harsh environmental conditions and low food supply at the cost of becoming physically inactive for periods lasting days, weeks, or months [5]. To ensure success, mammalian hibernators must increase food intake prior to entering hibernation to accumulate sufficient fat reserves which will later provide energy for day-to-day body maintenance and inducing arousal [5,6]. If too little fat is accumulated, individuals are in danger of depleting their reserves before the hibernation season is over [7,8,9]. In addition, survival during hibernation is also likely to be linked to nest quality [10] and local environmental conditions [11].

The West European hedgehog (*Erinaceus europaeus*) is a small (<1.5 kg) winter-hibernating mammal that is thought to be in decline in the UK [12,13]. The specific drivers of this decline are unclear, although a wide range of threats can be recognized, including the following: habitat loss, fragmentation, and degradation [14,15,16,17,18]; road traffic accidents [19,20,21]; the application of chemical herbicides, pesticides, and molluscicides, as well as the use of anticoagulant rodenticides [6,7,22]; competition with and predation by badgers (*Meles meles*) [23,24,25,26], and climate-driven changes in invertebrate prey availability and hibernation success [7].

Although timings differ in relation to climate, sex, body size, and condition, hedgehogs typically hibernate between November and April in the UK [6,7]. It is not unusual for hedgehogs to temporarily rouse during the hibernation period and active individuals may relocate to alternative nests [6,10,27]. These partial arousals can last anywhere from several hours to several days [7,28,29]. Since hedgehog hibernation timings are variable, it is difficult to pinpoint which factors trigger the process of entering and arousing from hibernation, although it is likely to involve environmental and hormonal cues related to lower ambient temperatures, shorter days, and reduced invertebrate prey availability [7].

Evidence suggests that hedgehogs are increasingly associated with areas of human habitation [26,30] with substantially higher densities observed in towns and cities than in rural habitats [31,32,33]. Despite a relative plethora of studies on the winter activity of captive, rehabilitated or rural-dwelling hedgehogs [9,10,27,28,29,34,35,36,37,38], our understanding of the behaviour of urban-dwelling hedgehogs during this period is limited [11].

Urban areas are associated with a range of factors that could potentially positively or negatively affect patterns of hibernation. For example, in addition to potential nesting sites in patches of remnant natural or semi-natural vegetation, hedgehogs can access cavities beneath buildings, gardens sheds, or decking within residential gardens; urban residents may also supply artificial refugia in the form of homemade or commercially available “hedgehog houses” [7,31]. However, within each of these habitats/locations, hedgehogs are exposed to different levels of disturbance from humans or companion animals [39,40], road traffic [19], and artificial light [41] and sound. Similarly, temperatures within different microhabitats are likely to vary in relation to, for example, the density and composition of surrounding buildings and associated structures [31,42]. It is possible that such ”urban-associated” factors could have direct impacts upon the onset of and patterns of arousal during hibernation. For example, warmer temperatures in urban areas [43] may stimulate early arousal from hibernation which, in turn, could increase fat consumption, thereby posing a risk to overwinter survival [7,11].

It has been suggested that supplementary feeding could, in particular, negatively affect natural patterns of hibernation behaviour in hedgehogs [44]. In the UK, many wildlife organisations actively encourage householders to leave out food for hedgehogs in gardens during the colder months in an effort to aid the accumulation of fat prior to hibernation but also to provide sustenance during periodic arousals when natural food availability is low (e.g., [45,46,47]). The effects of anthropogenic feeding on some aspects of the ecology of urban wildlife (e.g., density, health, and reproductive output) have been investigated extensively (e.g., [48,49,50]), but data on the impacts on hibernating species are limited. Key observations are that overwinter supplementary feeding is linked to the increased probability of sighting animals [51], interruptions to denning behaviour [52], and accelerated telomere attrition [53]. Conversely, artificial food sources could provide invaluable additional sustenance for individuals in need [6].

Overall, urban areas act as significant strongholds for the UK hedgehog population and expanding our knowledge of overwinter activity and the parameters affecting it is fundamental to developing robust conservation management strategies. Therefore, studies are needed which investigate the following: (a) the activity patterns of urban hedgehogs throughout the hibernation season and (b) how these are affected by external factors. In this study, we quantified patterns of hedgehog occupancy within residential gardens before, during, and after the winter season (see Methods for our definition of the winter season) in relation to within garden and surrounding habitat characteristics, environmental conditions (e.g., daylength and temperature), and patterns of anthropogenic feeding.

## 2. Materials and Methods

### 2.1. Footprint Tunnel Survey

Hedgehog presence/absence surveys were carried out in the back gardens of private households in the town of Reading, UK (51°, 27′ N and 0°, 58′ W; population >230,000; and area >60 km^2^) and its outskirts from 18 November 2017–7 April 2018. Badgers and foxes (*Vulpes vulpes*) which are both potential predators of hedgehogs and competitors for hedgehog food, are present in Reading, although records of the former indicate that they are limited to the northern section of the town [54]. Domestic dogs (*Canis familiaris*) were present in some gardens surveyed, but these were typically confined to the owner’s garden from approximately 11 p.m., whereas hedgehogs could be active throughout the night; consequently, dogs have been shown to not affect patterns of hedgehog occupancy [54]. Similarly, there has been no evidence to suggest that domestic cats (*Felis catus*) are likely to affect hedgehog occupancy either; cats pose little direct threat to hedgehogs and there is an abundance of anecdotal evidence of both species using the same garden at the same time.

Volunteers (citizen scientists) were recruited through an advert on social media in October 2017. Interested participants were asked to provide information on their garden location, current hedgehog-feeding habits, and, to the best of their knowledge, the frequency with which hedgehogs used their garden (ranging from “never” to “every night”). This information was used to categorise volunteers as those who were feeding hedgehogs prior to the start of the study itself (and, by default, who had hedgehogs in their garden), those who were not feeding hedgehogs at this time but who had them visiting their garden, and those who were not feeding hedgehogs at this time and who did not think they visited their garden. As we were interested in investigating the patterns of behaviour of hedgehogs in relation to the existing pattern of feeding by householders (i.e., this was an observational study), and because we were reliant on members of the public agreeing to participate, the distribution of households relative to one another and garden size were dependent on the volunteers themselves; these issues are considered further below.

Prior to the start of the study, householders that had been feeding hedgehogs were asked to either continue feeding them for the duration of the study (November–April) or to stop feeding completely; asking them to maintain a consistent pattern of feeding throughout the study simplified the analyses, especially as we had to assign the start and end of the hibernation period retrospectively. Consequently, the sample of householders consisted of the following four groups: (i) people that had been feeding hedgehogs previously and who continued to feed throughout the study, (ii) people that had been feeding hedgehogs previously but who stopped feeding for the duration of the study, (iii) householders that did not feed hedgehogs before and during the study but who did think they had hedgehogs in their garden, and (iv) householders that did not feed hedgehogs before and during the study and did not think they had hedgehogs in their garden. For those people that elected to continue feeding hedgehogs throughout the project, we asked that they carried on feeding at the same frequency, give the same volume of food each time, and not alter the type of food. This approach was adopted to avoid unduly affecting patterns of hedgehog behaviour in relation to changes in the amount of food available.

Gardens were surveyed using footprint tunnels, which have been used previously to survey hedgehogs in both rural and urban environments (e.g., [19,26,54,55,56]). Each householder was given one footprint tunnel and instructed to place the tunnel in their rear garden in a position where they thought hedgehogs would be likely to encounter it (e.g., parallel to fences at points where animals could enter the garden). Tunnels consisted of folded corrugated plastic in the form of a triangular tunnel (1200 × 210 × 180 mm) [55]. Ink (carbon powder mixed with vegetable oil) was applied to two strips of masking tape on either side of a food bait (~30 g of commercially available dry hedgehog food) in the centre of a removable plastic insert inside the tunnel; two sheets of A4 paper were fastened at either end of the insert to ”capture” footprints of any hedgehogs that traversed through. In order to attract animals without significantly influencing their behaviour, the pot containing the food was sealed but pierced with small holes to allow the scent of the bait to escape; this would prevent hedgehogs (and foxes or domestic cats) from depleting the food bait within a given survey period. Volunteers were given sufficient supplies for the footprint tunnel (e.g., food bait, ink, and paper) to last the duration of the study, as well as an instruction booklet and animal tracks identification guide.

Volunteers checked their tunnels every Saturday and submitted weekly presence/absence results of all tracks recorded through an online survey form (SurveyMonkey.com). Any suspected hedgehog footprints were photographed and sent digitally to one of the authors (AG) for verification. The study was terminated after 20 weeks when volunteer interest had started to decline (weekly reminders to prompt the submission of results needed to be increased markedly in the latter stages).

### 2.2. Dividing the Data into Seasons

Whilst it is understood that hibernation timings vary between individuals, we opted to subdivide the data into “seasons” that broadly reflected stages before, during, and after the principal hibernation period (henceforth denoted as autumn, winter, and spring). The purpose of this approach was to allow us to analyse the influence of different factors across the contrasting phases of the hibernation season when hedgehogs would be expected to place different emphasis on those factors. For example, the availability of anthropogenic food sources could be more important in the autumn season than the winter season, whereas the reverse could be true when considering access to a secure long-term nest site. Additionally, one assumption of occupancy analysis which we used to analyse these data was that sites remain closed to changes in occupancy between sampling visits [57]. This assumption would have been violated had the data been analysed as one continuous season as, for example, hedgehogs could have consistently used gardens during autumn and spring but not during winter.

The cut-off dates encompassing each season were informed by the pattern of occupancy observed during the 20-week survey. When ≤15% sites were occupied each week, the majority of hedgehogs were considered to be inactive and any data collected during that time were allocated to the winter category. Thus, the three time periods were identified as Weeks 1–7 (18 November 2017–05 January 2018), Weeks 8–16 (06 January 2018–09 March 2018), and Weeks 17–20 (10 March 2018–06 April 2018). Although we concede that this is an a posteriori approach to defining the hibernation period, the timing of low occupancy is in line with that reported elsewhere for hibernation in Britain at this latitude [7,10,21]. Analyses were, however, also conducted with an alternative cut-off threshold (≤20% sites occupied, Weeks 6–16) to investigate the consistency of the occupancy models; no marked differences in the results were evident (see Supplementary Table S1).

### 2.3. Data Analyses

Pearson Chi-squared tests were used initially to assess whether hedgehogs tended to be consistently present or absent in the same gardens between seasons. The effects of the variables listed in Table 1 on hedgehog presence/absence within each season were investigated using occupancy analysis, a technique which has been used successfully in previous studies of hedgehogs [26,54,55]. In occupancy modelling, an optimisation process is used to find the maximum likelihood of an event occurring. Data from each season were initially analysed independently of any covariates to identify whether the best-fitting baseline models were ones where weekly detection rates (*p*) were considered to be constant (detection probability did not vary between weeks within that season) or survey specific (detection probability did vary between weeks within that season). These initial analyses were also used to compare naïve occupancy (the proportion of sites where hedgehogs were detected) and true occupancy (Ψ, an estimate of the proportion of sites where hedgehogs were present, accounting for false absences). Analyses were conducted using Presence 12.24.

Variables were quantified using an online questionnaire at the end of the study, from the data itself or from external sources (Table 1). The questionnaire survey requested information about features within the participant’s back garden, the proportion of neighbouring gardens that were accessible to hedgehogs from their own garden, patterns of feeding during the study, and the number of potential nesting sites.

Three variables were used to investigate the potential effects of garden size and proximity to other survey gardens on patterns of detection and occupancy (Table 1). For example, garden size (mean ± SD = 238.5 ± 244.8 m^2^) could have potentially affected detection rates as we only used one footprint tunnel in each garden, although the majority of gardens (93.7%) covered <550 m^2^, three gardens (4.8%) covered 786–870 m^2^, and one garden (1.6%) was 1520 m^2^ in area. Within each season, the straight-line distance to the nearest other house and the straight-line distance to the nearest other house where hedgehogs were detected were incorporated to determine whether hedgehogs were more likely to be detected in houses close to one another, which would potentially indicate that patterns of detection were not independent.

Habitat characteristics in the area around each house were quantified using the straight-line distances to the nearest arable, grassland, and woodland habitats, and the total area of habitats within 250 and 500 m radii of each garden. These measures were quantified from Natural Environment Research Council land class datasets [58] with QGIS 3.4.4 (Table 1). Radii of 250 and 500 m were selected based upon existing data of hedgehog nightly ranges outside the hibernation season [15,59]. Minimum grass level and air temperatures, and weekly rainfall volume, were taken from a weather station on the University of Reading’s Whiteknights campus [60]. Mean weekly daylength was quantified from sunset and sunrise measurements from Benson weather station, approximately 18 km north of Reading [61]. As these data were taken from sites in proximity to the survey gardens, but not in the gardens themselves, they reflected general environmental conditions and not the specific micro-habitat characteristics of each garden.

Following checks for multicollinearity, single-species, single-season models were fitted; all variables were first considered in single-covariate models. Then, multi-covariate models were constructed based upon the known ecology of hedgehogs, as well as the hypothesised importance of different variables on occupancy during each season. Supplementary feeding before and during the study, as well as feeding intended for other species, were considered to be important in all seasons, and for autumn, models included the availability of and proximity to potential winter nesting sites; for winter and spring, models included environmental conditions that were likely to affect the timing of hibernation, i.e., daylength, ground temperature, and air temperature. A maximum of three covariates was considered in each model because of the relatively small sample sizes. This approach was favoured to produce a realistic set of candidate models, avoiding the shortcomings of algorithm-based model selection [62,63].

The goodness-of-fit of the most global model for each season was tested using the bootstrap method with 1000 replicates. Bootstrapping simulates detection histories for each site and produces a test statistic (Pearson Chi-squared) for each of the 1000 runs [64]. A measure of ”lack of fit”, defined as a variance inflation factor ĉ, is calculated by dividing the observed test statistic by the average bootstrap statistic [65]. When ĉ > 1, there is evidence of poor fit and it is recommended that (a) Akaike’s information criterion (AIC) values should be converted into quasi-likelihood adjusted AIC (QAIC) and (b) standard errors of beta estimates should be inflated by a factor of √ĉ [62,64,65]. Models that did not converge were excluded. Those with ΔQAIC values <2 were considered to be top-ranking models [62], and covariates were regarded as significant when their associated 95% confidence intervals did not cross 0 [66].

## 3. Results

### 3.1. General Trends

**Figure 1:**
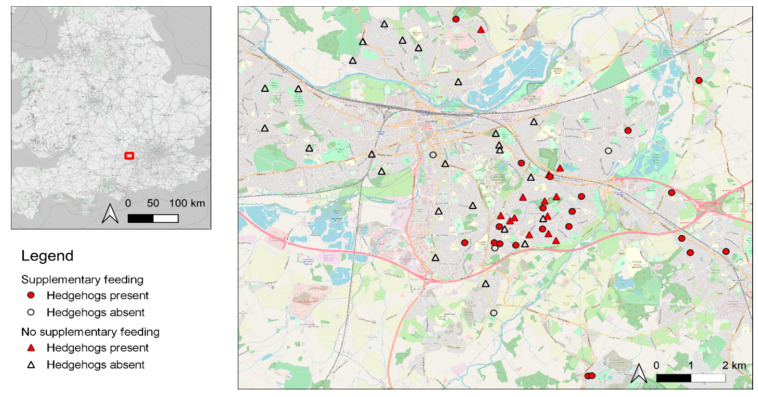
The locations of gardens (*n* = 63) in Reading and surrounding areas surveyed for hedgehogs between November 2017 and April 2018 inclusive. Circles denote gardens where hedgehogs were fed by householders prior to the study; diamonds denote gardens where hedgehogs were not fed prior to the study; filled and open symbols denote gardens where hedgehogs were and were not detected at any point during the current study, respectively.

Overall, 63 householders completed the study (Figure 1). During Week 1, results were obtained for 26 (41.2%) sites as compared with 100% in subsequent weeks; this was associated with the challenges of getting volunteers started but is not likely to have affected the results since occupancy analysis is robust to missing data [67]. In autumn, or ”pre-hibernation”, hedgehogs were and were not being fed in 25 (39.7%) and 38 (60.3%) gardens, respectively (Figure 1).

**Figure 2:**
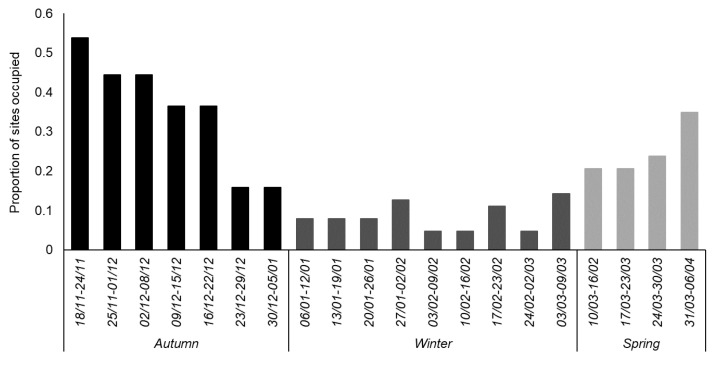
The proportion of all gardens surveyed (*n* = 26 in Week 1 and *n* = 63 for Weeks 2–20) where hedgehogs were recorded each week. Weekly survey dates are given in the format *dd/mm*, running from November 2017 to April 2018 inclusive.

Hedgehogs were active throughout all survey periods (Figure 2) and were recorded on 247 occasions (19.6% of the 1260 surveyor weeks). In autumn, hedgehogs were detected in 34 (54.0%) gardens, i.e., 21 of 25 (84.0%) gardens where they had been fed previously and 13 of 38 (34.2%) gardens where they had not been fed previously (Figure 1). Cumulatively, 97.1% of hedgehog-positive sites were detected by the third week of surveying. Occupancy (Ψ) and detection probability (*p*) were lowest between January and March (autumn true Ψ = 0.54, winter true Ψ = 0.32, and spring true Ψ = 0.39); full occupancy estimates from the baseline models are given in Table 2. False-absence error rates were very low (autumn 0.1%, winter 2.1%, and spring 0.6%).

Of the 34 hedgehog-positive gardens, 18 gardens (52.9%) were used every season, 9 (26.5%) were used during the autumn period only, and none were used exclusively during winter or spring. Consequently, there was a strong association in the pattern of presence/absence of hedgehogs in individual gardens between successive seasons, i.e., autumn-winter (Chi-squared test χ^2^_1_ = 23.204, *p* < 0.001) and winter-spring (χ^2^_1_ = 37.010, *p* < 0.001).

### 3.2. Factors Affecting Hedgehog Occupancy

For analyses incorporating covariates (Table 1), all top-ranking models included a feeding variable (Table 3). Occupancy in autumn and winter was associated with supplementary feeding prior to the hibernation period (FEDBEFORE), whereas in spring it was most associated with feeding in that season (FEEDHOG). There was also some support for detection probability being positively influenced by DAYTIME and FEEDOTHER during spring, but the effect was not significant. All other covariates reported in the best-fitting models, in each season, had statistically significant positive effects on occupancy or detection probability. Full model results can be found in Supplementary Tables S2–S4. Garden size, proximity to other gardens per se, and proximity to the nearest other garden where hedgehogs were detected in that season were not included in the top-ranked models in any season.

**Figure 3:**
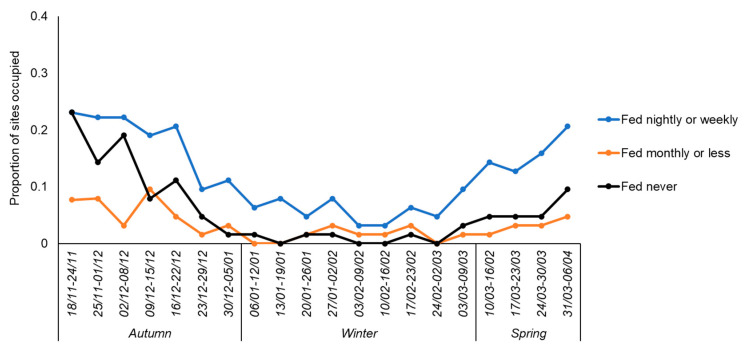
The proportion of gardens (*n* = 26 in Week 1 and *n* = 63 for Weeks 2–20) where hedgehogs were detected in relation to the frequency with which householders provided food at the outset of the study. Weekly survey dates are given in the format *dd/mm*, running from November 2017 to April 2018 inclusive.

In winter, hedgehogs were recorded in 16 of 25 (64.0%) gardens where the householder had been feeding them in autumn as compared with 3 of 38 (7.9%) gardens where they had not been fed. Overall, of the hedgehog-positive sites within each season, gardens where householders had previously put out food were visited, on average, for 4.4 weeks in autumn (*n* = 21 gardens, 62.9% of weeks in the 7-week season), 2.6 weeks in winter (*n* = 16 gardens, 28.9% of the nine-week season), and 2.7 weeks in spring (*n* = 18 gardens, 67.5% of the four-week season). Comparable figures for gardens where they were not fed were 3.3 weeks (*n* = 13 gardens, 47.1%), 2.0 weeks (*n* = 3 gardens, 22.2%), and 2.5 weeks (*n* = 6 gardens, 62.5%), respectively. Consequently, hedgehogs were much more likely to be present in gardens where food was supplied by householders (Figure 3).

## 4. Discussion

Hibernation is an adaptive physiological response to reduce energetic requirements during periods of low food availability. Hedgehogs, therefore, need to accumulate sufficient fat reserves prior to hibernation, and then minimize expenditure of energy during this period. In behavioural terms, this essentially means that hedgehogs need to avoid rousing unnecessarily from hibernation. However, they do need to retain the ability to be able to respond if environmental conditions become unfavourable or, for example, if they are detected by predators or disturbed. Consequently, individuals need to find locations that afford them protection, but which are also in proximity to alternative locations, with appropriate building materials, if they need to move.

In this study, hedgehog occupancy and detection in autumn were significantly linked to the area of woodland habitat within 500 m (WOOD 500 m) of focal gardens and the number of potential nest sites available within gardens (NESTSITES), respectively. Previous studies have reported that a significant proportion of winter nests are constructed in wooded areas [9,10] and the nearby woodland measured in this study area may have provided valuable pockets of semi-natural nesting habitat within an otherwise built-up area. However, the relative qualities of woodland and within-garden nesting sites are unknown. For example, wooded areas can be associated with a higher abundance of favoured building materials (the leaves of broadleaved trees: [7]) but urban woodlands are often open to the public and are likely to be associated with high levels of disturbance by walkers and especially their dogs. Alternatively, gardens offer potentially advantageous nesting sites such as beneath sheds and decking, but where natural nesting materials could be scarce. Future studies of urban hedgehog populations, therefore, need to focus on quantifying where hibernacula are located and if this is linked to over-winter survival rates.

Urban areas also pose one additional challenge. Research to date has indicated that hedgehogs tend to enter hibernation in response to the combination of a reduction in temperatures and a decline in food availability [7]. This was also evident in this study, with hedgehog detection during winter reduced as grass and air temperatures declined. In urban areas, however, food supplied by householders was not directly linked to prevailing temperatures. As a result, hedgehogs could be getting “mixed messages”, that is, food availability is still high even though temperatures are low. Ultimately, this could result in maladaptive responses leading to reduced over-winter survival rates and longevity.

In autumn, hedgehog occupancy was correlated with whether they had been fed in the previous season. Hedgehogs were detected in 54.0% of gardens overall, with a marked difference between those houses where they had (84.0%) and had not (34.2%) been fed. Similarly, occupancy in winter (30.2% of gardens overall) was also correlated with the pattern of feeding at the outset of the study, with an increase in the disparity between gardens where they had (64.0%) and had not been fed (7.9%). This was consistent with the radio-tracking data reported by Rasmussen et al. [11] which indicated that urban-dwelling hedgehogs tended to stay in the vicinity of local feeding stations during both active and inactive seasons, but also potentially suggested that patterns of feeding prior to hibernation could increase the likelihood that hedgehogs visited gardens during the hibernation period. In contrast, in spring, hedgehog occupancy tended to be associated with the frequency with which animals were being fed in that season, with occupancy higher where they were being fed more frequently.

Winter activity is, however, not unusual, and hedgehogs typically relocate nests at least once during the hibernation period [6,10,27]. As we used footprint tunnels to record hedgehog activity on a weekly basis, it was not possible to determine if detections during the winter season reflected individual animals in the normal process of relocating nests, or if they reflected the behaviour of several animals in the same garden. For example, the continued use of a single garden by individual hedgehogs overwinter has been recorded previously [9,11]. That being said, hedgehogs were detected for an average of 2.6 weeks in winter in gardens where they had been fed previously (*n* = 16) as compared with 2.0 weeks in other gardens (*n* = 3). Again, this is suggestive of the fact that householder feeding patterns could be influencing over-winter activity.

However, although anthropogenic feeding could negatively affect hedgehogs during hibernation [52,68], it is possible that it could be beneficial [9,28]. For example, it could enable animals that have not accumulated sufficient body fat to delay the point at which they enter hibernation [6], especially juveniles born in late summer [7]. Similarly, it could also help animals that have roused from hibernation to replenish some of their reserves. This could be important for animals that experience an increasing number of arousal events in relation to changing climatic conditions and anthropogenic influences.

Conversely, as hedgehogs are capable of surviving losses of up to 44% of their pre-hibernation weight [9,11,27,69], it is not clear if access to food during winter is beneficial. For example, it has been suggested that hedgehogs might only enter into a ”partial hibernation” where food is available [7]. Since even a single rousing can consume the same amount of energy required to survive 3–4 days of hibernation [36], animals can experience proportionately larger losses in mass overwinter if they cannot access sufficient food [7]. Furthermore, animals that are active during the winter also face the additional risks associated with, for example, road traffic, companion animals, and domestic gardens [11,21,39]. ”Shortenings” of the hibernation period, caused by increased arousals or a delay in hibernation commencement, have also been linked to accelerated cellular aging in mammals [53,70,71,72], thereby potentially having implications for longevity [73,74,75]. In addition, there is a need to consider the nutritional value of foods being provided by householders. For example, should animals come to rely on non-natural foods as a principal source of energy, it is possible that these do not fulfill their nutritional requirements and could compromise their condition [50]. Therefore, additional information is required on the types of food used by householders and its nutritional content relative to the needs of hedgehogs at this time.

Despite the absence of any definitive data that feeding hedgehogs overwinter is beneficial, it is encouraged by several wildlife organisations in Britain [45,46,47]. Given that arguments can be made that overwinter feeding could negatively impact hedgehogs, there is an urgent need to study its effects in more detail so that accurate advice can be given to householders. Such investigations will require the study of the activity and movement patterns, body mass changes, reproductive success, and longevity of individual hedgehogs before, during, and after the hibernation period in an experimental framework (i.e., controlling the frequency and volume of food supplied by randomly selected householders). These studies are, however, likely to be associated with significant challenges since they would require the cooperation of large numbers of householders for extended periods of time.

## 5. Conclusions

This study has indicated that residential gardens may be used frequently by hedgehogs throughout all stages of hibernation. Supplementary feeding in preceding seasons was found to be a key factor associated with hedgehog presence/absence during the hibernation period. This potentially indicates that supplementary feeding could affect key components of the hibernation behaviour of urban-dwelling hedgehogs, which could be detrimental or beneficial to overwinter survival and reproduction. Therefore, further intensive studies of known individuals before, during, and after the hibernation period are required.

## Figures and Tables

**Table 1:** Summary of the variables used in analysis, collected from questionnaire surveys and external data sources. Q indicates that the data were derived from a questionnaire survey of the householder; D indicates that the variable was extracted from the occupancy data itself; E indicates data from an external source (see text).

Covariate	Source	Description
FEEDHOG	Q	An ordinal measure of whether food was left out for hedgehogs during the study: 1 = never, 2 = less frequently (monthly or less), 3 = more frequently (nightly or weekly)
FEDBEFORE	Q	A binary measure of whether the participant usually left out food for hedgehogs prior to the commencement of the study
FEEDOTHERS	Q	A binary measure of whether food was left out by the participant for birds or other animals at some point during the study
NESTSITES	Q	The number of potential types of nest sites available in the participant’s garden as assessed by the participant. Tick-box options of possible nesting sites were listed on the questionnaire as “hedgehog house”, “under a shed or decking”, “under bushes or shrubs”, “under a compost heap” or “other (please provide more information)”. The total number of potential nest sites were converted to z-scores
CONNECTIVITY	Q	The proportion of front and back gardens neighbouring the participant’s household that is accessible for hedgehogs from the participant’s own gardens
FRONT2BACK	Q	A binary measure of whether a hedgehog could access the participant’s back garden from their front garden
GOODHABITAT	Q	The proportion of habitat in the participant’s back garden only that is considered ”good” for wildlife, including lawn, shrubs, flowerbeds and ponds
HOUSETYPE	Q	A binary measure of whether houses were: (i) semi-detached, link-detached or detached; or (ii) other (e.g., terraced)
GARDENSIZE	E	The area of each garden (m^2^) converted to z-scores
NEARESTOTHER	D	Distance from each site to the next nearest site (m) converted to z-scores
NEAREST + VE	D	Distance from each site to the next nearest hedgehog-positive site (m) per season (autumn, winter or spring) converted to z-scores
ARABLEDIST	E	Distance from each site to the nearest area of arable land (m) converted to z-scores
ARABLE 500 m	E	The area of arable land (m^2^) within a 500 m radius of each site converted to z-scores (Note: As only 4 sites fell within 250 m of arable land, the potential variable ARABLE250 m was not considered for analyses)
WOODDIST	E	Distance from each site to the nearest area of woodland (m) converted to z-scores
WOOD 250 m and WOOD 500 m	E	The area of woodland (m^2^) within 250 and 500 m radii of each site converted to z-scores
GRASSDIST	E	Distance from each site to the nearest area of grassland (m) converted to z-scores
GRASS 250 m and GRASS 500 m	E	The area of grassland (m^2^) within 250 and 500 m radii of each site converted to z-scores
URBAN 250 m and URBAN 500 m	E	The area of urban and suburban habitat (m^2^) within 250 and 500 m radii of each site converted to z-scores (Note: As all sites fell within the urban habitat classification, the straight-line distance from each site to urban habitat was not considered for analysis)
DAYTIME	E	Mean daylength (time between sunrise and sunset) per week, converted to z-scores
AIRTEMP	E	Minimum air temperature (°C) averaged per survey week based on hourly recordings taken between 21:00 and 09:00, converted to z-scores
GRASSTEMP	E	Minimum grass temperature (°C) averaged per survey week based on daily recordings taken at 09:00, converted to z-scores

**Table 2:** Summary of baseline hedgehog occupancy models where detection rate was modelled as constant (did not vary between weeks within each season) versus survey specific (did vary between weeks within each season). Seasons are illustrated in Figure 2.

Season	Model	QAIC	ΔQAIC	AIC Weight	Model Likelihood	*K*	Detection Rate	Naïve Ψ	True Ψ
Autumn	Ψ(.), *p*(survey-specific)	270.59	0.00	1	1.0000	8	0.8234	0.5397	0.5403
							0.8225		
							0.8225		
							0.6756		
							0.6756		
							0.2938		
							0.2938		
	Ψ(.), *p*(.)	294.26	23.67	0.0000	0.0000	2	0.6138	0.5397	0.5411
Winter	Ψ(.), *p*(.)	213.63	0.00	0.9852	1.0000	2	0.2626	0.3016	0.3224
	Ψ(.), *p*(survey-specific)	222.02	8.39	0.0148	0.0151	10	0.2481	0.3016	0.3198
							0.2481		
							0.2481		
							0.397		
							0.1489		
							0.1489		
							0.3474		
							0.1489		
							0.4467		
Spring	Ψ(.), *p*(.)	64.13	0.00	0.8006	1.0000	2	0.6459	0.3810	0.3870
	Ψ(.), *p*(survey-specific)	66.91	2.78	0.1994	0.2491	5	0.5377	0.3810	0.3838
							0.5377		
							0.6204		
							0.9100		

Ψ = occupancy, *p* = detection probability, *K* = number of parameters. ΔQAIC is the change in quasi-likelihood adjusted Akaike’s information criterion. For each season, the variance inflation factor ĉ was adjusted based on goodness-of-fit tests of the most parameterised models (1.3226, 1.3385, and 3.5534 for autumn, winter, and spring, respectively). Naïve occupancy is the number of gardens where hedgehogs were detected and true occupancy is the number of gardens estimated to be occupied by hedgehogs after accounting for the false-absence error rate.

**Table 3:** A summary of the top-ranking models (ΔQAIC < 2) produced in single-season occupancy analyses. Seasons are illustrated in Figure 2.

Season	Model	QAIC	ΔQAIC	AIC Weight	Model Likelihood	*K*
Autumn	Ψ(FEDBEFORE + WOOD500 m), *p*(survey + NESTSITES)	283.85	0.00	0.8966	1.0000	11
Winter	Ψ(FEDBEFORE), *p*(FEEDHOG + FEEDOTHERS)	214.42	0.00	0.4561	1.0000	5
	Ψ(FEDBEFORE), *p*(FEEDHOG + GRASSTEMP)	215.41	0.99	0.2780	0.6096	5
	Ψ(FEDBEFORE), *p*(FEEDHOG + AIRTEMP)	215.51	1.09	0.2645	0.5798	5
Spring	Ψ(FEEDHOG), *p*(DAYTIME + FEEDOTHERS)	71.83	0.00	0.2413	1.0000	5
	Ψ(FEDBEFORE), *p*(.)	71.97	0.14	0.2250	0.9324	3
	Ψ(FEEDHOG), *p*(.)	72.82	0.99	0.1471	0.6096	3

Ψ = occupancy, *p* = detection probability, *K* = number of parameters. ΔQAIC is the change in quasi-likelihood adjusted Akaike’s information criterion. For each season, the variance inflation factor ĉ was adjusted based on goodness-of-fit tests of the most parameterised models (1.1309, 1.1531, and 2.8138 for autumn, winter, and spring, respectively).

## Supplementary Materials

The following are available online at https://www.mdpi.com/2076-2615/10/8/1344/s1, Table S1: Model results of Weeks 6–16, Table S2–S4: Full occupancy model results.

## References

[1] Leather S.R., Walters K.F.A., Bale J.S. (1993). The Ecology of Insect Overwintering.

[2] Cáceres C.E. (1997). Dormancy in invertebrates. Invert. Biol..

[3] Wells K.D. (2007). The Ecology and Behavior of Amphibians.

[4] Ruf T., Geiser F. (2015). Daily torpor and hibernation in birds and mammals. Biol. Rev..

[5] Martin S.L., Yoder A.D. (2014). Theme and variations: Heterothermy in mammals. Integ. Comp. Biol..

[6] Reeve N. (1994). Hedgehogs.

[7] Morris P. (2018). Hedgehog.

[8] Kristiansson H. (1990). Population variables and causes of mortality in a hedgehog (*Erinaceus europaeus*) population in southern Sweden. J. Zool..

[9] Jensen A.B. (2004). Overwintering of European hedgehogs *Erinaceus europaeus* in a Danish rural area. Acta Theriol..

[10] Morris P. (1973). Winter nests of the hedgehog (*Erinaceus europaeus L*.). Oecologia.

[11] Rasmussen S.L., Berg T.B., Dabelsteen T., Jones O.R. (2019). The ecology of suburban juvenile European hedgehogs (*Erinaceus europaeus*) in Denmark. Ecol. Evol..

[12] Roos S., Johnston A., Noble D. (2012). UK Hedgehog Datasets and Their Potential for Long-Term Monitoring. BTO Research Report, 598.

[13] Mathews F., Kubasiewicz L., Gurnell J., Harrower C., McDonald R., Shore R. (2018). A Review of the Population and Conservation Status of British Mammals. A Report by the Mammal Society under Contract to Natural England, Natural Resources Wales and Scottish Natural Heritage.

[14] Becher S.A., Griffiths R. (1998). Genetic differentiation among local populations of the European hedgehog (*Erinaceus europaeus*) in mosaic habitats. Mol. Ecol..

[15] Rondinini C., Doncaster C. (2002). Roads as barriers to movement for hedgehogs. Funct. Ecol..

[16] Hof A.R., Bright P.W. (2009). The value of green-spaces in built-up areas for western hedgehogs. Lutra.

[17] Hof A.R., Bright P.W. (2010). The value of agri-environment schemes for macro-invertebrate feeders: Hedgehogs on arable farms in Britain. Anim. Conserv..

[18] Moorhouse T.P., Palmer S.C.F., Travis J.M.J., Macdonald D.W. (2014). Hugging the hedges: Might agri-environment manipulations affect landscape permeability for hedgehogs?. Biol. Conserv..

[19] Huijser M.P., Bergers P.J.M. (2000). The effect of roads and traffic on hedgehog (*Erinaceus europaeus*) populations. Biol. Cons..

[20] Wembridge D.E., Newman M.R., Bright P.W., Morris P.A. (2016). An estimate of the annual number of hedgehog (*Erinaceus europaeus*) road casualties in Great Britain. Mamm. Comm..

[21] Wright P.G.R., Coomber F.G., Bellamy C.C., Perkins S.E., Mathews F. (2020). Predicting hedgehog mortality risks on British roads using habitat suitability modelling. PeerJ.

[22] Dowding C.V., Shore R.F., Worgan A., Baker P.J., Harris S. (2010). Accumulation of anticoagulant rodenticides in a non-target insectivore, the European hedgehog (*Erinaceus europaeus*). Environ. Pollut..

[23] Young R.P., Davison J., Trewby I.D., Wilson G.J., Delahay R.J., Doncaster C.P. (2006). Abundance of hedgehogs (*Erinaceus europaeus*) in relation to the density and distribution of badgers (*Meles meles*). J. Zool..

[24] Trewby I.D., Young R., McDonald R.A., Wilson G.J., Davison J., Walker N., Robertson A., Doncaster C.P., Delahay R.J. (2014). Impacts of removing badgers on localised counts of hedgehogs. PLoS ONE.

[25] Pettett C.E., Moorhouse T.P., Johnson P.J., Macdonald D.W. (2018). Factors affecting hedgehog (*Erinaceus europaeus*) attraction to rural villages in arable landscapes. Eur. J. Wildl. Res..

[26] Williams B., Baker P.J., Thomas E., Wilson G., Judge J., Yarnell R.W. (2018). Reduced occupancy of hedgehogs (*Erinaceus europaeus*) in rural England and Wales: The influence of habitat and an asymmetric intraguild predator. Sci. Rep..

[27] Yarnell R.W., Surgery J., Grogan A., Thompson R., Davies K., Kimbrough C., Scott D.M. (2019). Should rehabilitated hedgehogs be released in winter? A comparison of survival, nest use and weight change in wild and rescued animals. Eur. J. Wildl. Res..

[28] Walhovd H. (1979). Partial arousals from hibernation in hedgehogs in outdoor hibernacula. Oecologia.

[29] Webb P.I., Ellison J. (1998). Normothermy, torpor, and arousal in hedgehogs (*Erinaceus europaeus*) from Dunedin, New Zealand. J. Zool..

[30] Doncaster C.P. (1994). Factors regulating local variations in abundance: Field tests on hedgehogs, *Erinaceus europaeus*. Oikos.

[31] Hubert P., Julliard R., Biagianti S., Poulle M.L. (2011). Ecological factors driving the higher hedgehog (*Erinaceus europeaus*) density in an urban area compared to the adjacent rural area. Landsc. Urban Plan..

[32] Van de Poel J.L., Dekker J., van Langevelde F. (2015). Dutch hedgehogs *Erinaceus europaeus* are nowadays mainly found in urban areas, possibly due to the negative effects of badgers *Meles meles*. Wild. Biol..

[33] Schaus J., Uzal A., Gentle L.K., Baker P.J., Bearman-Brown L., Bullion S., Gazzard A., Lockwood H., North A., Reader T. (2020). Application of the Random Encounter Model in citizen science projects to monitor animal densities. Remote Sens. Ecol. Cons..

[34] Soivio A., Tähti H., Kristoffersson R. (1968). Studies on the periodicity of hibernation in the hedgehog (*Erinaceus europaeus L.*): III. Hibernation in a constant ambient temperature of −5° C. Ann. Zoo. Fenn..

[35] Parkes J. (1975). Some aspects of the biology of the hedgehog (*Erinaceus europaeus L.*) in the Manawatu, New Zealand. New Zeal. J. Zool..

[36] Tähti H., Soivio A. (1977). Respiratory and circulatory differences between induced and spontaneous arousals in hibernating hedgehogs (*Erinaceus europaeus L*.). Ann. Zool. Fenn..

[37] Dmi’el R., Schwarz M. (1984). Hibernation patterns and energy expenditure in hedgehogs from semi-arid and temperate habitats. J. Comp. Physiol. B.

[38] Fowler P.A., Racey P.A. (1984). Daily and seasonal cycles of body temperature and aspects of heterothermy in the hedgehog *Erinaceus europaeus*. J. Comp. Physiol. B.

[39] Stocker L. (1987). The Complete Hedgehog.

[40] Rast W., Barthel L.M.F., Berger A. (2019). Music festival makes hedgehogs move: How individuals cope behaviorally in response to human-induced stressors. Animals.

[41] Finch D., Smith B.R., Marshall C., Coomber F.G., Kubasiewicz L.M., Anderson M., Wright P.G.R., Mathews F. (2020). Effects of Artificial Light at Night (ALAN) on European hedgehog activity at supplementary feeding stations. Animals.

[42] Perini K., Magliocco A. (2014). Effects of vegetation, urban density, building height, and atmospheric conditions on local temperatures and thermal comfort. Urban For. Urban Green..

[43] Chapman S., Watson J.E., Salazar A., Thatcher M., McAlpine C.A. (2017). The impact of urbanization and climate change on urban temperatures: A systematic review. Landsc. Ecol..

[44] Keep Feeding Hedgehogs in the Autumn—University of Brighton. https://www.brighton.ac.uk/about-us/news-and-events/news/2017/09-13-keep-feeding-hedgehogs-in-the-autumn.aspx.

[45] Hedgehog Street: Should I Keep Feeding Hedgehogs Overwinter?. https://www.hedgehogstreet.org/should-i-keep-feeding-hedgehogs-over-winter/.

[46] British Hedgehog Preservation Society: Feeding. https://www.britishhedgehogs.org.uk/feeding/.

[47] Tiggywinkles Wildlife Hospital Hedgehog Fact Sheet. https://www.sttiggywinkles.org.uk/hedgehog-fact-sheet/.

[48] Robb G.N., McDonald R.A., Chamberlain D.E., Bearhop S. (2008). Food for thought: Supplementary feeding as a driver of ecological change in avian populations. Front. Ecol. Environ..

[49] Ewen J.G., Walker L., Canessa S., Groombridge J.J. (2014). Improving supplementary feeding in species conservation. Conserv. Biol..

[50] Murray M.H., Becker D.J., Hall R.J., Hernandez S.M. (2016). Wildlife health and supplemental feeding: A review and management recommendations. Biol. Conserv..

[51] Bojarskaa K., Drobniak S., Jakubiec Z., Zyśk-Gorczyńsk E. (2019). Winter insomnia: How weather conditions and supplementary feeding affect the brown bear activity in a long-term study. Glob. Ecol. Conserv..

[52] Krofel M., Spacapan M., Jerina K. (2017). Winter sleep with room service: Denning behaviour of brown bears with access to anthropogenic food. J. Zool..

[53] Kirby R., Johnson H.E., Alldredge M.W., Pauli J.N. (2019). The cascading effects of human food on hibernation and cellular aging in free-ranging black bears. Sci. Rep..

[54] Williams B., Mann N., Neumann J.L., Yarnell R.W., Baker P.J. (2018). A prickly problem: Developing a volunteer-friendly tool for monitoring populations of a terrestrial urban mammal, the West European hedgehog (*Erinaceus europaeus*). Urban Ecosyst..

[55] Yarnell R.W., Pacheco M., Williams B., Neumann J.L., Rymer D.J., Baker P.J. (2014). Using occupancy analysis to validate the use of footprint tunnels as a method for monitoring the hedgehog *Erinaceus europaeus*. Mamm. Rev..

[56] Williams R.L., Stafford R., Goodenough A. (2014). Biodiversity in urban gardens: Assessing the accuracy of citizen science data on garden hedgehogs. Urban Ecosyst..

[57] MacKenzie D.I., Royle J.A. (2005). Designing occupancy studies: General advice and allocating survey effort. J. Appl. Ecol..

[58] Land Cover Map 2015 (Vector, GB). https://catalogue.ceh.ac.uk/documents/6c6c9203-7333-4d96-88ab-78925e7a4e73.

[59] Dowding C.V., Harris S., Poulton S., Baker P.J. (2010). Nocturnal ranging behaviour of urban hedgehogs, *Erinaceus europaeus*, in relation to risk and reward. Anim. Behav..

[60] Met Office Integrated Data Archive System (MIDAS) Land and Marine Surface Stations Data (1853-Current). https://catalogue.ceda.ac.uk/uuid/220a65615218d5c9cc9e4785a3234bd0.

[61] Reading, England, United Kingdom—Sunrise, Sunset, and Daylength. https://www.timeanddate.com/sun/uk/reading.

[62] Burnham K.P., Anderson D.R. (1998). Model Selection and Inference.

[63] Whittingham M.J., Stephens P.A., Bradbury R.B., Freckleton R.P. (2006). Why do we still use stepwise modelling in ecology and behaviour?. J. Anim. Ecol..

[64] Mackenzie D.I., Bailey L.L. (2004). Assessing the fit of site-occupancy models. JABES.

[65] Program MARK: A Gentle Introduction. http://www.phidot.org/software/mark/docs/book/.

[66] Exercises in Occupancy Modelling and Estimation Exercise 4: Single-Species, Single-Season Model with Site Level Covariates. http://www.uvm.edu/rsenr/vtcfwru/spreadsheets/occupancy/occupancy.htm.

[67] Mackenzie D.I., Nichols J.D., Royle J.A., Pollock K.H., Bailey L.L., Hines J.E. (2006). Occupancy Estimation and Modeling Inferring Patterns and Dynamics of Species Occurrence.

[68] Baldwin R.A., Bender L.C. (2010). Denning chronology of black bears in eastern rocky mountain national park, Colorado. West. North Am. Nat..

[69] Haigh A., O’Riordan R.M., Butler F. (2012). Nesting behaviour and seasonal body mass changes in a rural Irish population of the Western hedgehog (*Erinaceus europaeus*). Acta Theirol..

[70] Turbill C., Smith S., Deimel C., Ruf T. (2012). Daily torpor is associated with telomere length change over winter in Djungarian hamsters. Biol. Lett..

[71] Turbill C., Ruf T., Smith S., Bieber C. (2013). Seasonal variation in telomere length of a hibernating rodent. Biol. Lett..

[72] Hoelzl F., Cornils J.S., Smith S., Moodley Y., Ruf T. (2016). Telomere dynamics in free-living edible dormice (*Glis glis*): The impact of hibernation and food supply. J. Exp. Biol..

[73] Lyman C.P., Brien R.C.O., Greene G.C., Papafrangos E.D. (1981). Hibernation and longevity in the Turkish hamster *Mesocricetus brandi*. Science.

[74] Blanco M.B., Zehr S.M. (2015). Striking longevity in a hibernating lemur. J. Zool..

[75] Wu C., Storey K.B. (2016). Life in the cold: Links between mammalian hibernation and longevity. Biomol. Concepts.

