# Supplementary material for: Patterns of Feeding by Householders Affect Activity of Hedgehogs (Erinaceus europaeus) during the Hibernation Period
Source: Animals (Basel). 2020 Aug 4;10(8):1344. doi: 10.3390/ani10081344 (PMC7460126; doi:10.3390/ani10081344)
Supplement: Supplementary file 1 [file animals-10-01344-s001.pdf]

Table S1. Full occupancy models for winter season based upon alternative cut-off threshold of < 20% of gardens occupied per week. Under this scenario, the winter season would have been defined as Weeks 6-16 (23/12/2017–09/03/2018) rather than Weeks 8-16 (06/01/2018-09/03/2018) as presented in the manuscript. Detection probability was modelled as constant. The variance inflation factor ĉ was adjusted based on goodness-of-fit tests of one of the most parameterised models (2.5817). The three top models in this extended timeframe (11 weeks) are the same as those presented in the manuscript (9 weeks).

Model	QAIC	ΔQAIC	AIC weight	Model likelihood	*K*	-2*LogLike	Covariates	Estimate	SE	Inflated SE	Lower limit	Upper limit
psi(FEDBEFORE),p(FEEDHOG + GRASSTEMP)	136.82	0.00	0.3436	1.0000	5	327.41	psi(FEDBEFORE)	2.408	0.730	1.173	0.109	4.707
							p(FEEDHOG)	1.089	0.275	0.442	0.223	1.955
							p(GRASSTEMP)	0.501	0.177	0.285	-0.057	1.060
psi(FEDBEFORE),p(FEEDHOG + AIRTEMP)	137.08	0.26	0.3017	0.8781	5	328.09	psi(FEDBEFORE)	2.408	0.730	1.173	0.108	4.708
							p(FEEDHOG)	1.087	0.275	0.441	0.222	1.952
							p(AIRTEMP)	0.489	0.181	0.291	-0.081	1.059
psi(FEDBEFORE),p(FEEDHOG + FEEDOTHERS)	138.76	1.94	0.1303	0.3791	5	332.41	psi(FEDBEFORE)	2.628	0.834	1.340	0.002	5.255
							p(FEEDHOG)	0.919	0.279	0.448	0.042	1.796
							p(FEEDOTHERS)	1.398	0.670	1.077	-0.712	3.508
psi(.),p(FEDBEFORE)	140.14	3.32	0.0653	0.1901	3	346.32	p(FEDBEFORE)	2.373	0.445	0.715	0.972	3.773
psi(.),p(FEEDHOG)	141.12	4.30	0.0400	0.1165	3	348.85	p(FEEDHOG)	1.293	0.290	0.465	0.380	2.205
psi(FEDBEFORE),p(.)	142.34	5.52	0.0217	0.0633	3	352.00	psi(FEDBEFORE)	2.300	0.629	1.010	0.320	4.281
psi(FEEDHOG),p(.)	143.95	7.13	0.0097	0.0283	3	356.15	psi(FEEDHOG)	1.187	0.385	0.619	-0.026	2.400
psi(.),p(GARDENSIZE)	144.11	7.29	0.0090	0.0261	3	356.56	p(GARDENSIZE)	-1.007	0.363	0.584	-2.151	0.137
psi(.),p(FEEDOTHERS)	144.71	7.89	0.0066	0.0194	3	358.10	p(FEEDOTHERS)	1.906	0.638	1.026	-0.104	3.917
psi(NEAREST+VE),p(.)	144.90	8.08	0.0060	0.0176	3	358.60	psi(NEAREST+VE)	-1.171	0.499	0.802	-2.742	0.400
psi(.),p(GRASSTEMP)	145.00	8.18	0.0058	0.0167	3	358.86	p(GRASSTEMP)	0.487	0.175	0.281	-0.063	1.038
psi(.),p(AIRTEMP)	145.26	8.44	0.0051	0.0147	3	359.52	p(AIRTEMP)	0.476	0.179	0.287	-0.087	1.038
1 group, Constant P	146.43	9.61	0.0028	0.0082	2	367.70						
psi(.),p(RAINFALL)	146.60	9.78	0.0026	0.0075	3	362.98	p(RAINFALL)	0.322	0.149	0.239	-0.146	0.791
psi(.),p(ARABLE500m)	146.73	9.91	0.0024	0.0070	3	363.32	p(ARABLE500m)	0.249	0.117	0.188	-0.119	0.618
psi(.),p(GOODHABITAT)	146.91	10.09	0.0022	0.0064	3	363.78	p(GOODHABITAT)	-1.741	0.865	1.389	-4.464	0.982
psi(GARDENSIZE),p(.)	147.08	10.26	0.0020	0.0059	3	364.22	psi(GARDENSIZE)	-0.703	0.457	0.734	-2.141	0.736
psi(.),p(URBAN500m)	147.11	10.29	0.0020	0.0058	3	364.30	p(URBAN500m)	-0.318	0.170	0.274	-0.855	0.218
psi(WOODDIST),p(.)	147.14	10.32	0.0020	0.0057	3	364.38	psi(WOODDIST)	-0.543	0.319	0.513	-1.548	0.463
psi(CONNECTIVITY),p(.)	147.32	10.50	0.0018	0.0052	3	364.84	psi(CONNECTIVITY)	1.684	1.034	1.661	-1.572	4.939

psi(GRASS250m),p(.)	147.45	10.63	0.0017	0.0049	3	365.17	psi(GRASS250m)	-0.520	0.375	0.603	-1.701	0.661
psi(WOOD500m),p(.)	147.45	10.63	0.0017	0.0049	3	365.19	psi(WOOD500m)	0.434	0.280	0.451	-0.449	1.317
psi(FEEDOTHERS),p(.)	147.62	10.80	0.0016	0.0045	3	365.63	psi(FEEDOTHERS)	0.976	0.717	1.152	-1.281	3.233
psi(.),p(GRASS250m)	147.66	10.84	0.0015	0.0044	3	365.73	p(GRASS250m)	0.412	0.277	0.445	-0.461	1.286
psi(.),p(HOUSETYPE)	147.67	10.85	0.0015	0.0044	3	365.74	p(HOUSETYPE)	0.604	0.455	0.731	-0.829	2.037
psi(.),p(ARABLEDIST)	147.72	10.90	0.0015	0.0043	3	365.87	p(ARABLEDIST)	-0.222	0.168	0.271	-0.752	0.308
psi(.),p(GRASS500m)	147.73	10.91	0.0015	0.0043	3	365.91	p(GRASS500m)	0.291	0.210	0.337	-0.370	0.951
psi(.),p(NEARESTOTHER)	147.82	11.00	0.0014	0.0041	3	366.13	psi(NEARESTOTHER)	0.144	0.114	0.183	-0.213	0.502
psi(GRASS500m),p(.)	147.91	11.09	0.0013	0.0039	3	366.38	psi(GRASS500m)	-0.339	0.314	0.505	-1.329	0.650
psi(GOODHABITAT),p(.)	147.94	11.12	0.0013	0.0038	3	366.44	psi(GOODHABITAT)	1.849	1.732	2.783	-3.607	7.304
psi(.),p(DAYTIME)	147.94	11.12	0.0013	0.0038	3	366.45	p(DAYTIME)	-0.169	0.152	0.244	-0.648	0.310
psi(.),p(CONNECTIVITY)	148.06	11.24	0.0012	0.0036	3	366.76	p(CONNECTIVITY)	0.565	0.602	0.967	-1.331	2.461
psi(URBAN250m),p(.)	148.13	11.31	0.0012	0.0035	3	366.93	psi(URBAN250m)	0.247	0.292	0.469	-0.672	1.167
psi(.),p(NESTSITES)	148.13	11.31	0.0012	0.0035	3	366.94	p(NESTSITES)	0.126	0.145	0.234	-0.332	0.584
psi(FRONT2BACK),p(.)	148.19	11.37	0.0012	0.0034	3	367.08	psi(FRONT2BACK)	-0.424	0.543	0.873	-2.135	1.288
psi(ARABLE500m),p(.)	148.22	11.40	0.0011	0.0033	3	367.18	psi(ARABLE500m)	0.189	0.263	0.423	-0.640	1.018
psi(.),p(NEAREST+VE)	148.31	11.49	0.0011	0.0032	3	367.40	p(NEAREST+VE)	-0.462	0.434	0.698	-1.830	0.906
psi(WOOD250m),p(.)	148.32	11.50	0.0011	0.0032	3	367.43	psi(WOOD250m)	0.137	0.266	0.428	-0.702	0.975
psi(NESTSITES),p(.)	148.34	11.52	0.0011	0.0032	3	367.48	psi(NESTSITES)	0.125	0.271	0.435	-0.727	0.978
psi(.),p(GRASSDIST)	148.35	11.53	0.0011	0.0031	3	367.51	p(GRASSDIST)	0.066	0.153	0.247	-0.417	0.549
psi(URBAN500m),p(.)	148.36	11.54	0.0011	0.0031	3	367.52	psi(URBAN500m)	0.116	0.279	0.448	-0.761	0.994
psi(.),p(WOODDIST)	148.37	11.55	0.0011	0.0031	3	367.56	p(WOODDIST)	-0.081	0.216	0.347	-0.762	0.599
psi(.),p(URBAN250m)	148.37	11.55	0.0011	0.0031	3	367.55	p(URBAN250m)	-0.069	0.187	0.300	-0.657	0.520
psi(GRASSDIST),p(.)	148.38	11.56	0.0011	0.0031	3	367.59	psi(GRASSDIST)	0.092	0.270	0.434	-0.760	0.943
psi(.),p(FRONT2BACK)	148.38	11.56	0.0011	0.0031	3	367.58	p(FRONT2BACK)	-0.100	0.313	0.503	-1.085	0.885
psi(.),p(WOOD250m)	148.40	11.58	0.0011	0.0031	3	367.63	p(WOOD250m)	-0.042	0.167	0.269	-0.569	0.486
psi(NEARESTOTHER),p(.)	148.41	11.59	0.0010	0.0030	3	367.67	psi(NEARESTOTHER)	0.042	0.267	0.429	-0.799	0.882
psi(ARABLEDIST),p(.)	148.41	11.59	0.0010	0.0030	3	367.65	psi(ARABLEDIST)	-0.057	0.273	0.439	-0.917	0.802
psi(.),p(WOOD500m)	148.42	11.60	0.0010	0.0030	3	367.68	p(WOOD500m)	-0.020	0.165	0.266	-0.541	0.501
psi(HOUSETYPE),p(.)	148.42	11.60	0.0010	0.0030	3	367.68	psi(HOUSETYPE)	0.088	0.690	1.108	-2.084	2.261

Table S2. Full occupancy model results for autumn (Weeks 1-7: 18/11/17-05/01/2018). Detection probability was modelled as survey-specific [p(survey)].

Model	QAIC	ΔQAIC	AIC weight	Model likelihood	*K*	-2*LogLike	Covariates	Estimate	SE	Inflated SE	Lower limit	Upper limit
psi(FEDBEFORE + WOOD500m),p(survey + NESTSITES)	283.85	0.00	0.8966	1.0000	11	296.13	psi(FEDBEFORE)	2.771	0.792	0.843	1.119	4.423
							psi(WOOD500m)	1.308	0.432	0.459	0.407	2.208
							p(NESTSITES)	0.456	0.161	0.171	0.122	0.791
psi(FEDBEFORE + WOOD500m),p(survey)	289.43	5.58	0.0551	0.0614	10	304.70	psi(FEDBEFORE)	2.730	0.769	0.818	1.127	4.334
							psi(WOOD500m)	1.290	0.421	0.447	0.413	2.167
psi(WOOD500m),p(survey + FEEDHOG)	290.47	6.62	0.0327	0.0365	10	305.87	psi(WOOD500m)	1.088	0.365	0.388	0.328	1.848
							p(FEEDHOG)	0.872	0.236	0.251	0.380	1.364
psi(NEAREST+VE),p(survey)	292.12	8.27	0.0143	0.0160	9	310.00	psi(NEAREST+VE)	-2.288	0.693	0.737	-3.732	-0.843
psi(FEDBEFORE),p(survey)	299.15	15.30	0.0004	0.0005	9	317.95	psi(FEDBEFORE)	2.316	0.646	0.687	0.969	3.664
psi(.),p(survey + FEEDHOG)	299.44	15.59	0.0004	0.0004	9	318.28	p(FEEDHOG)	0.873	0.239	0.254	0.374	1.372
psi(FEEDHOG),p(survey)	301.97	18.12	0.0001	0.0001	9	321.14	psi(FEEDHOG)	1.153	0.347	0.369	0.430	1.876
psi(WOOD500m),p(survey)	302.48	18.63	0.0001	0.0001	9	321.72	psi(WOOD500m)	1.038	0.336	0.358	0.338	1.739
psi(.),p(survey + NEARESTOTHER)	303.15	19.30	0.0001	0.0001	9	322.48	p(NEARESTOTHER)	0.633	0.218	0.232	0.179	1.088
psi(.),p(survey + FEDBEFORE)	303.45	19.60	0.0000	0.0001	9	322.81	p(FEDBEFORE)	1.118	0.345	0.367	0.399	1.836
psi(.),p(survey + GRASS250m)	303.93	20.08	0.0000	0.0000	9	323.36	p(GRASS250m)	1.325	0.447	0.476	0.393	2.257
psi(WOODDIST),p(survey)	305.61	21.76	0.0000	0.0000	9	325.26	psi(WOODDIST)	-0.850	0.322	0.342	-1.521	-0.179
psi(.),p(survey + NESTSITES)	305.89	22.04	0.0000	0.0000	9	325.57	p(NESTSITES)	0.456	0.161	0.172	0.120	0.792
psi(.),p(survey + CONNECTIVITY)	308.45	24.60	0.0000	0.0000	9	328.47	p(CONNECTIVITY)	1.277	0.547	0.582	0.136	2.417
psi(.),p(survey + GRASS500M)	308.51	24.66	0.0000	0.0000	9	328.54	p(GRASS500M)	0.526	0.231	0.246	0.044	1.007
psi(.),p(survey + URBAN500m)	308.69	24.84	0.0000	0.0000	9	328.74	p(URBAN500m)	-0.449	0.200	0.213	-0.866	-0.032
psi(GRASS250m),p(survey)	308.85	25.00	0.0000	0.0000	9	328.92	psi(GRASS250m)	-0.656	0.330	0.351	-1.344	0.033
psi(.),p(survey + NEAREST+VE)	309.12	25.27	0.0000	0.0000	9	329.23	p(NEAREST+VE)	0.987	0.475	0.505	-0.003	1.977
psi(CONNECTIVITY),p(survey)	309.18	25.33	0.0000	0.0000	9	329.30	psi(CONNECTIVITY)	2.023	0.958	1.019	0.025	4.020
psi(.),p(survey + FRONT2BACK)	310.09	26.24	0.0000	0.0000	9	330.32	p(FRONT2BACK)	0.614	0.320	0.340	-0.053	1.281
psi(NEARESTOTHER),p(survey)	310.11	26.26	0.0000	0.0000	9	330.35	psi(NEARESTOTHER)	-0.534	0.299	0.318	-1.156	0.089
psi(.),p(survey + URBAN250m)	310.29	26.44	0.0000	0.0000	9	330.55	p(URBAN250m)	-0.349	0.191	0.203	-0.747	0.049
psi(WOOD250m),p(survey)	310.42	26.57	0.0000	0.0000	9	330.70	psi(WOOD250m)	0.549	0.343	0.365	-0.166	1.263
psi(GARDENAREA),p(survey)	310.62	26.77	0.0000	0.0000	9	330.92	psi(GARDENAREA)	-0.520	0.332	0.353	-1.213	0.172
psi(.),p(survey + GOODHABITAT)	311.02	27.17	0.0000	0.0000	9	331.38	p(GOODHABITAT)	1.311	0.802	0.853	-0.361	2.983

1 group, Survey-specific P.	311.41	27.56	0.0000	0.0000	8	334.08					
psi(.),p(survey + GRASSDIST)	311.62	27.77	0.0000	0.0000	9	332.05	p(GRASSDIST)	-0.239	0.168	0.179	-0.590	0.112
psi(.),p(survey + ARABLE500m)	311.84	27.99	0.0000	0.0000	9	332.30	p(ARABLE500m)	0.216	0.166	0.177	-0.130	0.562
psi(.),p(survey + ARABLEDIST)	312.04	28.19	0.0000	0.0000	9	332.53	p(ARABLEDIST)	0.211	0.171	0.182	-0.146	0.567
psi(GRASS500m),p(survey)	312.40	28.55	0.0000	0.0000	9	332.94	psi(GRASS500m)	-0.275	0.264	0.280	-0.825	0.274
psi(.),p(survey + WOODDIST)	312.41	28.56	0.0000	0.0000	9	332.95	p(WOODDIST)	-0.241	0.234	0.248	-0.728	0.246
psi(.),p(survey + HOUSETYPE)	312.69	28.84	0.0000	0.0000	9	333.27	p(HOUSETYPE)	0.370	0.411	0.437	-0.487	1.226
psi(.),p(survey + GARDENAREA)	312.72	28.87	0.0000	0.0000	9	333.30	p(GARDENAREA)	-0.196	0.233	0.248	-0.681	0.290
psi(.),p(survey + FEEDOTHERS)	312.76	28.91	0.0000	0.0000	9	333.34	p(FEEDOTHERS)	0.327	0.381	0.406	-0.468	1.122
psi(NESTSITES),p(survey)	312.93	29.08	0.0000	0.0000	9	333.54	psi(NESTSITES)	0.188	0.258	0.275	-0.350	0.726
psi(FEEDOTHERS),p(survey)	313.03	29.18	0.0000	0.0000	9	333.65	psi(FEEDOTHERS)	0.386	0.594	0.632	-0.853	1.625
psi(ARABLEDIST),p(survey)	313.10	29.25	0.0000	0.0000	9	333.73	psi(ARABLEDIST)	-0.150	0.256	0.272	-0.684	0.384
psi(URBAN250m),p(survey)	313.12	29.27	0.0000	0.0000	9	333.75	psi(URBAN250m)	0.145	0.256	0.272	-0.388	0.678
psi(.),p(survey + WOOD250m)	313.14	29.29	0.0000	0.0000	9	333.77	p(WOOD250m)	0.074	0.134	0.143	-0.206	0.354
psi(GRASSDIST),p(survey)	313.23	29.38	0.0000	0.0000	9	333.87	psi(GRASSDIST)	0.116	0.257	0.273	-0.420	0.651
psi(.),p(survey + WOOD500m)	313.23	29.38	0.0000	0.0000	9	333.88	p(WOOD500m)	0.071	0.158	0.168	-0.259	0.401
psi(HOUSETYPE),p(survey)	313.32	29.47	0.0000	0.0000	9	333.98	psi(HOUSETYPE)	0.197	0.643	0.684	-1.143	1.537
psi(GOODHABITAT),p(survey)	313.37	29.52	0.0000	0.0000	9	334.03	psi(GOODHABITAT)	-0.301	1.444	1.535	-3.310	2.708
psi(FRONT2BACK),p(survey)	313.40	29.55	0.0000	0.0000	9	334.07	psi(FRONT2BACK)	-0.048	0.507	0.539	-1.105	1.008
psi(.),p(survey + RAINFALL)	313.41	29.56	0.0000	0.0000	9	334.08	p(RAINFALL)	-0.627	22.009	23.405	-46.501	45.247
psi(.),p(survey + AIRTEMP)	313.41	29.56	0.0000	0.0000	9	334.08	p(AIRTEMP)	0.143	4.183	4.449	-8.577	8.863
psi(.),p(survey + DAYTIME)	313.41	29.56	0.0000	0.0000	9	334.08	p(DAYTIME)	0.578	69.709	74.132	-144.720	145.876
psi(.),p(survey + GRASSTEMP)	313.41	29.56	0.0000	0.0000	9	334.08	p(GRASSTEMP)	-0.111	30.739	32.689	-64.182	63.960
psi(URBAN500m),p(survey)	313.41	29.56	0.0000	0.0000	9	334.08	psi(URBAN500m)	-0.010	0.255	0.272	-0.542	0.523
psi(ARABLE500m),p(survey)	343.05	59.20	0.0000	0.0000	3	381.17	psi(ARABLE500m)	0.075	0.259	0.275	-0.464	0.614

Table S3. Full occupancy model results for winter (Weeks 8-16: 06/01/2018-09/03/2018). Detection probability was modelled as constant.

Model	QAIC	ΔQAIC	AIC weight	Model likelihood	*K*	-2*LogLike	Covariates	Estimate	SE	Inflated SE	Lower limit	Upper limit
psi(FEDBEFORE),p(FEEDHOG + FEEDOTHERS)	214.42	0.00	0.4561	1.0000	5	235.72	psi(FEDBEFORE)	3.674	1.099	1.180	1.362	5.986
							p(FEEDHOG)	1.168	0.359	0.385	0.413	1.924
							p(FEEDOTHERS)	1.913	0.794	0.853	0.241	3.586
psi(FEDBEFORE),p(FEEDHOG + GRASSTEMP)	215.41	0.99	0.2780	0.6096	5	236.86	psi(FEDBEFORE)	3.196	0.901	0.968	1.299	5.093
							p(FEEDHOG)	1.277	0.368	0.395	0.503	2.052
							p(GRASSTEMP)	0.440	0.201	0.216	0.018	0.863
psi(FEDBEFORE),p(FEEDHOG + AIRTEMP)	215.51	1.09	0.2645	0.5798	5	236.97	psi(FEDBEFORE)	3.196	0.901	0.968	1.299	5.093
							p(FEEDHOG)	1.277	0.368	0.395	0.502	2.051
							p(AIRTEMP)	0.444	0.207	0.222	0.009	0.878
psi(FEDBEFORE),p(.)	226.65	12.23	0.0010	0.0022	3	254.43	psi(FEDBEFORE)	3.157	0.772	0.829	1.533	4.781
psi(.),p(FEEDHOG)	229.40	14.98	0.0003	0.0006	3	257.60	p(FEEDHOG)	1.714	0.372	0.400	0.931	2.497
psi(FEEDHOG),p(.)	232.95	18.53	0.0000	0.0001	3	261.70	psi(FEEDHOG)	1.609	0.477	0.512	0.605	2.614
psi(.),p(FEDBEFORE)	233.80	19.38	0.0000	0.0001	3	262.68	p(FEDBEFORE)	2.540	0.513	0.551	1.460	3.620
psi(.),p(GARDENSIZE)	234.66	20.24	0.0000	0.0000	3	263.67	p(GARDENSIZE)	-1.829	0.565	0.606	-3.017	-0.640
psi(.),p(FEEDOTHERS)	239.51	25.09	0.0000	0.0000	3	269.26	p(FEEDOTHERS)	2.326	0.826	0.887	0.587	4.065
psi(NEAREST+VE),p(.)	240.59	26.17	0.0000	0.0000	3	270.51	psi(NEAREST+VE)	-1.113	0.522	0.560	-2.211	-0.016
psi(GARDENSIZE),p(.)	241.65	27.23	0.0000	0.0000	3	271.73	psi(GARDENSIZE)	-1.245	0.653	0.701	-2.618	0.129
psi(.),p(NEAREST+VE)	242.03	27.61	0.0000	0.0000	3	272.17	p(NEAREST+VE)	-0.997	0.396	0.425	-1.830	-0.163
psi(.),p(GRASSTEMP)	242.56	28.14	0.0000	0.0000	3	272.78	p(GRASSTEMP)	0.430	0.198	0.213	0.013	0.848
psi(.),p(AIRTEMP)	242.66	28.24	0.0000	0.0000	3	272.89	p(AIRTEMP)	0.434	0.204	0.219	0.004	0.863
psi(FEEDOTHERS),p(.)	244.44	30.02	0.0000	0.0000	3	274.95	psi(FEEDOTHERS)	1.299	0.829	0.890	-0.446	3.043
psi(.),p(CONNECTIVITY)	244.88	30.46	0.0000	0.0000	3	275.45	p(CONNECTIVITY)	1.118	0.747	0.802	-0.455	2.690
psi(.),p(GOODHABITAT)	244.97	30.55	0.0000	0.0000	3	275.56	p(GOODHABITAT)	-1.635	1.043	1.120	-3.830	0.560
1 group, Constant P	245.01	30.59	0.0000	0.0000	2	277.91						
psi(.),p(ARABLE500m)	245.27	30.85	0.0000	0.0000	3	275.90	p(ARABLE500m)	0.198	0.137	0.147	-0.091	0.486
psi(.),p(FRONT2BACK)	245.45	31.03	0.0000	0.0000	3	276.11	p(FRONT2BACK)	-0.531	0.403	0.433	-1.379	0.318
psi(GRASS250m),p(.)	245.52	31.10	0.0000	0.0000	3	276.19	psi(GRASS250m)	-0.445	0.381	0.409	-1.246	0.356
psi(WOOD500m),p(.)	245.57	31.15	0.0000	0.0000	3	276.25	psi(WOOD500m)	0.367	0.290	0.311	-0.242	0.977
psi(HOUSETYPE),p(.)	245.80	31.38	0.0000	0.0000	3	276.51	psi(HOUSETYPE)	0.937	0.843	0.905	-0.837	2.712

psi(.),p(WOODDIST)	245.94	31.52	0.0000	0.0000	3	276.67	p(WOODDIST)	-0.335	0.313	0.336	-0.993	0.322
psi(WOODDIST),p(.)	245.94	31.52	0.0000	0.0000	3	276.67	psi(WOODDIST)	-0.287	0.251	0.269	-0.814	0.241
psi(ARABLE500m),p(.)	246.03	31.61	0.0000	0.0000	3	276.78	psi(ARABLE500m)	0.289	0.275	0.295	-0.289	0.867
psi(NESTSITES),p(.)	246.09	31.67	0.0000	0.0000	3	276.85	psi(NESTSITES)	0.293	0.288	0.310	-0.314	0.900
psi(CONNECTIVITY),p(.)	246.14	31.72	0.0000	0.0000	3	276.91	psi(CONNECTIVITY)	1.025	1.044	1.121	-1.172	3.222
psi(.),p(HOUSETYPE)	246.16	31.74	0.0000	0.0000	3	276.93	p(HOUSETYPE)	-0.588	0.564	0.605	-1.775	0.598
psi(NEARESTOTHER),p(.)	246.23	31.81	0.0000	0.0000	3	277.01	psi(NEARESTOTHER)	0.260	0.277	0.298	-0.323	0.844
psi(URBAN250m),p(.)	246.30	31.88	0.0000	0.0000	3	277.09	psi(URBAN250m)	0.273	0.314	0.337	-0.387	0.934
psi(GRASS500m),p(.)	246.47	32.05	0.0000	0.0000	3	277.29	psi(GRASS500m)	-0.239	0.317	0.341	-0.907	0.429
psi(.),p(URBAN250m)	246.55	32.13	0.0000	0.0000	3	277.38	p(URBAN250m)	0.181	0.255	0.274	-0.355	0.717
psi(.),p(DAYTIME)	246.66	32.24	0.0000	0.0000	3	277.50	p(DAYTIME)	0.114	0.178	0.191	-0.261	0.488
psi(ARABLEDIST),p(.)	246.68	32.26	0.0000	0.0000	3	277.53	psi(ARABLEDIST)	-0.179	0.294	0.316	-0.798	0.441
psi(FRONT2BACK),p(.)	246.72	32.30	0.0000	0.0000	3	277.57	psi(FRONT2BACK)	-0.328	0.571	0.613	-1.529	0.873
psi(.),p(NEARESTOTHER)	246.73	32.31	0.0000	0.0000	3	277.59	p(NEARESTOTHER)	-0.086	0.154	0.166	-0.411	0.239
psi(.),p(RAINFALL)	246.75	32.33	0.0000	0.0000	3	277.61	p(RAINFALL)	0.096	0.176	0.188	-0.274	0.465
psi(.),p(WOOD500m)	246.78	32.36	0.0000	0.0000	3	277.64	p(WOOD500m)	-0.097	0.188	0.201	-0.491	0.298
psi(GOODHABITAT),p(.)	246.83	32.41	0.0000	0.0000	3	277.70	psi(GOODHABITAT)	0.744	1.680	1.804	-2.791	4.280
psi(GRASSDIST),p(.)	246.84	32.42	0.0000	0.0000	3	277.71	psi(GRASSDIST)	0.127	0.283	0.304	-0.470	0.723
psi(.),p(GRASSDIST)	246.88	32.46	0.0000	0.0000	3	277.76	p(GRASSDIST)	0.068	0.177	0.190	-0.306	0.441
psi(.),p(URBAN500m)	246.90	32.48	0.0000	0.0000	3	277.78	p(URBAN500m)	-0.078	0.214	0.230	-0.528	0.372
psi(.),p(GRASS500m)	246.91	32.49	0.0000	0.0000	3	277.79	p(GRASS500m)	-0.107	0.317	0.341	-0.775	0.561
psi(.),p(WOOD250m)	246.92	32.50	0.0000	0.0000	3	277.81	p(WOOD250m)	-0.080	0.254	0.273	-0.615	0.454
psi(.),p(ARABLEDIST)	246.96	32.54	0.0000	0.0000	3	277.85	p(ARABLEDIST)	-0.043	0.187	0.201	-0.437	0.350
psi(WOOD250m),p(.)	246.99	32.57	0.0000	0.0000	3	277.89	psi(WOOD250m)	-0.042	0.292	0.313	-0.656	0.572
psi(URBAN500m),p(.)	247.00	32.58	0.0000	0.0000	3	277.90	psi(URBAN500m)	0.016	0.287	0.309	-0.589	0.621
psi(.),p(NESTSITES)	247.00	32.58	0.0000	0.0000	3	277.90	p(NESTSITES)	-0.015	0.182	0.195	-0.397	0.367
psi(.),p(GRASS250m)	247.01	32.59	0.0000	0.0000	3	277.91	p(GRASS250m)	0.020	0.598	0.642	-1.239	1.278

Table S4. Full occupancy model results for spring (Weeks 17-20: 10/03/2018-06/04/2018). Detection probability was modelled as constant.

Model	QAIC	ΔQAIC	AIC weight	Model likelihood	*K*	-2*LogLike	Covariates	Estimate	SE	Inflated SE	Lower limit	Upper limit
psi(FEEDHOG),p(DAYTIME + FEEDOTHER)	71.83	0.00	0.2413	1.0000	5	173.97	psi(FEEDHOG)	1.555	0.422	0.708	0.168	2.943
							p(DAYTIME)	0.758	0.278	0.467	-0.158	1.674
							p(FEEDOTHER)	1.356	0.550	0.923	-0.453	3.166
psi(FEDBEFORE),p(.)	71.97	0.14	0.2250	0.9324	3	185.63	psi(FEDBEFORE)	2.657	0.644	1.081	0.538	4.776
psi(FEEDHOG),p(.)	72.82	0.99	0.1471	0.6096	3	188.02	psi(FEEDHOG)	1.546	0.418	0.701	0.172	2.921
psi(NEAREST+VE),p(.)	76.13	4.30	0.0281	0.1165	3	197.32	psi(NEAREST+VE)	-1.183	0.503	0.844	-2.837	0.470
psi(.),p(FRONT2BACK)	76.35	4.52	0.0252	0.1044	3	197.94	p(FRONT2BACK)	-1.365	0.481	0.806	-2.945	0.216
psi(.),p(NEAREST+VE)	76.41	4.58	0.0244	0.1013	3	198.13	p(NEAREST+VE)	-1.410	0.468	0.786	-2.949	0.130
psi(.),p(DAYTIME)	76.67	4.84	0.0215	0.0889	3	198.85	p(DAYTIME)	0.710	0.267	0.449	-0.169	1.590
psi(.),p(FEEDOTHER)	77.28	5.45	0.0158	0.0655	3	200.58	p(FEEDOTHER)	1.277	0.532	0.892	-0.471	3.026
1 group, Constant P	77.41	5.58	0.0148	0.0614	2	206.57						
psi(WOOD500m),p(.)	77.53	5.70	0.0140	0.0578	3	201.27	psi(WOOD500m)	0.626	0.285	0.478	-0.311	1.563
psi(.),p(AIRTEMP)	78.29	6.46	0.0095	0.0396	3	203.42	p(AIRTEMP)	0.252	0.143	0.239	-0.217	0.721
psi(.),p(GRASSTEMP)	78.46	6.63	0.0088	0.0363	3	203.90	p(GRASSTEMP)	0.257	0.159	0.267	-0.266	0.779
psi(ARABLEDIST),p(.)	78.47	6.64	0.0087	0.0362	3	203.93	psi(ARABLEDIST)	-0.454	0.293	0.492	-1.418	0.509
psi(.),p(FEEDHOG)	78.52	6.69	0.0085	0.0353	3	204.06	p(FEEDHOG)	1.084	0.427	0.716	-0.319	2.487
psi(WOODDIST),p(.)	78.58	6.75	0.0083	0.0342	3	204.22	psi(WOODDIST)	-0.432	0.296	0.496	-1.405	0.541
psi(GRASS250m),p(.)	78.75	6.92	0.0076	0.0314	3	204.70	psi(GRASS250m)	-0.410	0.329	0.552	-1.492	0.671
psi(ARABLE500m),p(.)	78.80	6.97	0.0074	0.0307	3	204.85	psi(ARABLE500m)	0.344	0.271	0.455	-0.547	1.236
psi(URBAN250m),p(.)	78.85	7.02	0.0072	0.0299	3	204.98	psi(URBAN250m)	0.355	0.295	0.495	-0.614	1.324
psi(.),p(GRASS250m)	79.00	7.17	0.0067	0.0277	3	205.42	p(GRASS250m)	0.526	0.492	0.826	-1.093	2.144
psi(GARDENSIZE),p(.)	79.07	7.24	0.0065	0.0268	3	205.60	psi(GARDENSIZE)	0.001	0.004	0.007	-0.014	0.015
psi(.),(GARDENSIZE)	79.07	7.24	0.0065	0.0268	3	205.61	p(GARDENSIZE)	0.001	0.013	0.021	-0.041	0.043
psi(.),p(GRASS500m)	79.08	7.25	0.0064	0.0266	3	205.62	p(GRASS500m)	0.282	0.292	0.489	-0.677	1.241
psi(CONNECTIVITY),p(.)	79.11	7.28	0.0063	0.0263	3	205.72	psi(CONNECTIVITY)	0.869	0.953	1.599	-2.266	4.004
psi(.),p(GRASSDIST)	79.11	7.28	0.0063	0.0263	3	205.73	p(GRASSDIST)	-0.186	0.204	0.341	-0.855	0.483
psi(.),p(URBAN500m)	79.17	7.34	0.0061	0.0255	3	205.89	p(URBAN500m)	-0.204	0.250	0.419	-1.024	0.617
psi(NESTSSITES),p(.)	79.17	7.34	0.0061	0.0255	3	205.89	psi(NESTSITES)	0.217	0.266	0.446	-0.658	1.092
psi(GRASSDIST),p(.)	79.18	7.35	0.0061	0.0253	3	205.91	psi(GRASSDIST)	0.213	0.265	0.445	-0.659	1.085

psi(.),p(RAINFALL)	79.19	7.36	0.0061	0.0252	3	205.93	p(RAINFALL)	0.198	0.251	0.421	-0.626	1.023
psi(.),p(FEDBEFORE)	79.19	7.36	0.0061	0.0252	3	205.94	p(FEDBEFORE)	0.497	0.673	1.130	-1.717	2.711
psi(URBAN500m),p(.)	79.22	7.39	0.0060	0.0248	3	206.04	psi(URBAN500m)	-0.190	0.263	0.442	-1.056	0.675
psi(.),p(ARABLE500m)	79.23	7.40	0.0060	0.0247	3	206.05	p(ARABLE500m)	0.139	0.198	0.333	-0.513	0.792
psi(.),p(WOOD250m)	79.26	7.43	0.0059	0.0244	3	206.13	p(WOOD250m)	-0.234	0.363	0.609	-1.429	0.960
psi(GOODHABITAT),p(.)	79.26	7.43	0.0059	0.0244	3	206.15	psi(GOODHABITAT)	-0.950	1.468	2.463	-5.777	3.876
psi(.),p(ARABLEDIST)	79.27	7.44	0.0058	0.0242	3	206.17	p(ARABLEDIST)	-0.147	0.236	0.396	-0.923	0.629
psi(.),p(HOUSETYPE)	79.28	7.45	0.0058	0.0241	3	206.20	p(HOUSETYPE)	0.329	0.546	0.916	-1.466	2.124
psi(WOOD250m),p(.)	79.30	7.47	0.0058	0.0239	3	206.26	psi(WOOD250m)	-0.152	0.281	0.471	-1.076	0.772
psi(.),p(CONNECTIVITY)	79.33	7.50	0.0057	0.0235	3	206.33	p(CONNECTIVITY)	0.406	0.835	1.400	-2.338	3.150
psi(.),p(WOOD500m)	79.36	7.53	0.0056	0.0232	3	206.41	p(WOOD500m)	-0.086	0.219	0.368	-0.806	0.635
psi(.),p(URBAN250m)	79.37	7.54	0.0056	0.0231	3	206.45	p(URBAN250m)	0.106	0.324	0.543	-0.958	1.171
psi(FRONT2BACK),p(.)	79.38	7.55	0.0055	0.0229	3	206.48	psi(FRONT2BACK)	0.156	0.525	0.880	-1.569	1.881
psi(HOUSETYPE),p(.)	79.38	7.55	0.0055	0.0229	3	206.48	psi(HOUSETYPE)	-0.187	0.660	1.108	-2.358	1.985
psi(.),p(WOODDIST)	79.38	7.55	0.0055	0.0229	3	206.49	p(WOODDIST)	0.101	0.342	0.573	-1.023	1.225
psi(.),p(GOODHABITAT)	79.39	7.56	0.0055	0.0228	3	206.51	p(GOODHABITAT)	0.229	0.973	1.633	-2.971	3.429
psi(.),p(NEARESTOTHER)	79.39	7.56	0.0055	0.0228	3	206.50	p(NEARESTOTHER)	0.048	0.189	0.318	-0.575	0.670
psi(GRASS500m),p(.)	79.40	7.57	0.0055	0.0227	3	206.54	psi(GRASS500m)	-0.039	0.267	0.448	-0.916	0.839
psi(.),p(NESTSITES)	79.40	7.57	0.0055	0.0227	3	206.54	p(NESTSITES)	-0.042	0.229	0.384	-0.795	0.710
psi(FEEDOTHER),p(.)	79.40	7.57	0.0055	0.0227	3	206.53	psi(FEEDOTHER)	-0.106	0.612	1.026	-2.118	1.905
psi(NEARESTOTHER),p(.)	79.41	7.58	0.0055	0.0226	3	206.56	psi(NEARESTOTHER)	-0.018	0.264	0.443	-0.887	0.851
